# PPP1R14B Is a Prognostic and Immunological Biomarker in Pan-Cancer

**DOI:** 10.3389/fgene.2021.763561

**Published:** 2021-11-11

**Authors:** Mingxia Deng, Long Peng, Jiamin Li, Xiong Liu, Xichun Xia, Guangqiang Li

**Affiliations:** ^1^ Biomedical Translational Research Institute, The First Affiliated Hospital, Jinan University, Guangzhou, China; ^2^ Department of Cardiovascular Medicine, The Third Affiliated Hospital, Sun Yat-Sen University, Guangzhou, China; ^3^ Department of General Surgery, Dongguan Tungwah Hospital, Dongguan, China

**Keywords:** PPP1R14B, pan-cancer, prognosis, tumor infiltration, MDSC

## Abstract

Recent studies have shown that PPP1R14B was highly expressed in tumor tissues and patients with high expression of PPP1R14B had poor survival rates. However, the function and mechanisms of PPP1R14B in tumor progression remain ill defined. There was also lack of pan-cancer evidence for the relationship between PPP1R14B and various tumor types based on abundant clinical data. We used the TCGA project and GEO databases to perform pan-cancer analysis of PPP1R14B, including expression differences, correlations between expression levels and survival, genetic alteration, immune infiltration, and relevant cellular pathways, to investigate the functions and potential mechanisms of PPP1R14B in the pathogenesis or clinical prognosis of different cancers. Herein, we found that PPP1R14B was involved in the prognosis of pan-cancer and closely related to immune infiltration. Increased PPP1R14B expression correlated with poor prognosis and increased immune infiltration levels in myeloid-derived suppressor cells (MDSCs). Our studies suggest that PPP1R14B can be used as a prognostic biomarker for pan-cancer. Our findings may provide an antitumor strategy targeting PPP1R14B, including manipulation of tumor cell growth or the tumor microenvironment, especially myeloid-derived suppressor cell infiltration.

## Introduction

Protein phosphatase 1 (PP1) is an ubiquitous Ser-/Thr-specific phosphatase that regulates various cellular processes and has multiple functions in cellular processes, including RNA splicing, protein synthesis, cell cycle progression, and glycogen metabolism (Choy et al., 2012). PP1 can promote the rational use of energy, relax actomyosin fibers, restore the basic mode of protein synthesis, and regulate the recycling of transcription and splicing factors. PP1 plays a key role in stress recovery but promotes apoptosis when cells are damaged and cannot be repaired. In addition, it participates in neuronal excitement and ion channel transport. Finally, PP1 promotes the exit of mitosis and maintains the cell cycle in the G1 or G2 phase (Ceulemans and Bollen, 2004).

The PPP1R14B (protein phosphatase 1 regulatory inhibitor subunit 14B) gene, was first isolated as a novel gene close to the human phosphoinositide-specific phospholipase C beta 3 gene (PLCB3) on chromosomal region 11q13 (Lagercrantz et al., 1996). The PPP1R14B protein is capable of inhibiting PP1 as well as different PP1 holoenzymes. So, it is also named phosphatase holoenzyme inhibitor 1 (PHI-1) (Eto et al., 1999). The PPP1R14B gene is also known as PLCB3N, SOM172, and PNG.

PPP1R14B, localized in the juxtamembrane of the smooth muscle cells (Tountas et al., 2004), participates in regulating the trailing edge of migrating cells and modulates the retraction of endothelial and epithelial cells (Tountas and Brautigan, 2004). A study showed that the 3-gene expression signatures composed of SCGB2A1, KLF4, and PPP1R14B can differentiate a group of circa 5% of cases with short survival in chronic lymphocytic leukemia (CLL) patients, which is useful for further studies regarding disease prognostication and drug response in CLL (Orgueira et al., 2019). PPP1R14B was significantly overexpressed in ovarian clear cell carcinoma (OCCC) and associated endometriosis (Worley et al., 2015). A bioinformatics analysis showed that PPP1R14B was significantly overexpressed in plasma mRNAs in PCa (prostate cancer) patients, but the role of PPP1R14B in cancer was still unknown (Wang et al., 2020). Recent studies also showed that PPP1R14B were highly expressed in tumor tissues and patients with high expression of PPP1R14B had poor survival rates (Zhao et al., 2021). However, the function and mechanisms of PPP1R14B in tumor progression remain ill-defined. There is also no pan-cancer evidence for the relationship between PPP1R14B and various tumor types based on abundant clinical data.

The tumor microenvironment (TME) contains many infiltrated immune cells. The interaction between tumor cells and immune cells regulates the initiation and progression of tumors (Quail and Joyce, 2013). In the process of tumor initiation and progression, the immune system mainly goes through the following three processes: immune monitoring, immune balance, and immune destruction. The immune system presents a unique duality. Immune cells play a natural role at the beginning of tumor invasion, antitumor effect, and abnormally become a tumor-promoting phenotype in the process of tumor progression, assisting tumor immune escape and distant metastasis (Swann and Smyth, 2007). Immune cells in the TME infiltrate and secrete inflammatory mediators to form an inflammatory microenvironment with a high degree of heterogeneity. Immune cell components are complex and diverse, including T lymphocytes, B lymphocytes, macrophages of the innate immune defense system, natural killer (NK) cells, and dendritic cells (DC) with antigen-presenting effects (Giraldo et al., 2019). Among them, regulatory T cells (Tregs), tumor-associated macrophages (TAMs), and myeloid-derived suppressor cells (MDSCs) mediate immunosuppression and escort the development of tumors (Haist et al., 2021). The TME plays an important role in tumor progression, immune escape, and drug resistance. Thus, it is necessary to explore the characteristics of the TME, immune conditions, and related immunotherapy strategies for further clarifying the regulatory mechanism of the immune microenvironment to achieve precise immune intervention on tumors.

Herein, we used the TCGA project and GEO databases to perform pan-cancer analysis of PPP1R14, including expression differences, correlations between expression levels and survival, genetic alteration, immune infiltration, and relevant cellular pathways, to investigate the functions and potential mechanisms of PPP1R14B in the pathogenesis or clinical prognosis of different cancers.

## Materials and Methods

### Gene and Protein Expression Analysis

Tumor Immune Estimation Resource (TIMER, http://timer.cistrome.org/) is a comprehensive resource for systematic analysis of immune infiltration across diverse cancer types, including gene expression, clinical outcomes, somatic mutations, somatic copy number alterations, and the characterization of the tumor-immune system. The database includes 10,897 samples across 32 cancer types from TCGA to estimate the abundance of immune infiltration (Li et al., 2017; Li et al., 2020). We input PPP1R14B in the “Gene_DE” module of TIMER2.0 web and analyzed the expression difference of PPP1R14B between tumor and adjacent normal tissues for the 32 cancer types derived from the TCGA project.

GEPIA (http://gepia.cancer-pku.cn/) is a web-based tool to deliver fast and customizable functions, including differential expression analysis, correlation analysis, patient survival analysis, similar gene detection, and dimensionality reduction analysis (Tang et al., 2017; Tang et al., 2019). We analyzed the expression of PPP1R14B across TCGA tumors, and the matched TCGA normal and GTEx data were included as controls.

UALCAN (http://ualcan.path.uab.edu), an interactive web-portal, uses TCGA RNA-seq and clinical data from 31 cancer types and can be easily used for in-depth analyses of TCGA gene expression data (Chandrashekar et al., 2017). Herein, we analyzed the relative expression of PPP1R14B across tumor and normal samples, as well as in various tumor models based on individual tumor grade. The protein expression was analyzed using the data from the Clinical Proteomic Tumor Analysis Consortium (CPTAC, http://ualcan.path.uab.edu/analysis-prot.html).

### Survival Prognosis Analysis

The GEPIA2.0 web-based tool was used flor patient survival analysis. We input the PPP1R14B gene in the “survival analysis” module, selected a custom cancer type, and used the log-rank test for overall survival analysis. High-expression and low-expression cohorts were splitted by the high (50%) and low (50%) cutoff values.

The Kaplan–Meier plotter (http://kmplot.com/analysis/) is capable of assessing the effect of 54k genes (mRNA, miRNA, and protein) on survival in 21 cancer types. Sources of the databases include GEO, EGA, and TCGA (Nagy et al., 2021). We analyzed the correlation between PPP1R14B expression and survival in different cancer types in the “pan-cancer RNA-seq” module.

### Genetic Alteration Analysis

The cBioPortal web (https://www.cbioportal.org/) was used for genetic alteration analysis (Cerami et al., 2012). We chose the “curated set of non-redundant studies” (184 studies, 48,035 samples) in the “query” section and entered “PPP1R14B” for queries about the genetic alteration characteristics of PPP1R14B. The results of the alteration frequency, mutation type, and CNA (copy number alteration) across all tumors were observed in the “cancer types summary” module. Then, the data of the PP1R14B mRNA expression level vs. putative copy number alterations (mRNA VS CNA) were obtained in the “plots” module and then analyzed by using Graphpad software. The mutated site information of PPP1R14B can be displayed in the “mutations” module. We also used the “comparison/survival” module to obtain the data on the overall and relapse-free survival differences for the cancer cases with or without PPP1R14B genetic alteration.

### Immune Infiltration Analysis

We used the TIMER2.0 (http://timer.cistrome.org/) resource for systematic analysis of immune infiltrates across diverse cancer types (Li et al., 2020). We input the PPP1R14B gene in the “gene” module in the “immune” section and then selected the immune infiltrate type to obtain the data about the correlation of its expression with the immune infiltration level in diverse cancer types. We analyzed the important immune infiltrating cells, including B cells, CD4^+^ T cells, CD8^+^ T cells, Tregs, Tfh cells, γδ T cells, monocytes, macrophages, neutrophils, DC, and MDSCs.

### PPP1R14B-Related Gene Enrichment Analysis

We used the STRING website (https://string-db.org/) to obtain the PPP1R14B-binding proteins for protein–protein interaction network functional enrichment analysis (von Mering et al., 2003). We searched PPP1R14B in the query of “Protein by name” and chose “*Homo sapiens*”. Subsequently, we set the following main parameters: meaning of network edges (“evidence”), active interaction sources (“textmining”, “experiments”, “databases”, “co expression”, “neighborhood”, “gene fusion”, and “co-occurrence”), network type (“full network”), minimum required interaction score [“medium confidence (0.400)”], and maximum number of interactors to show (“no more than 20 interactors” in 1st shell).

We used TIMER2.0 to explore the correlation between PPP1R14B with a list of genes of the 20 interactors of PPP1R14B-binding in various cancer types. We input the PPP1R14B gene in the “Gene_Corr” section in the “exploration” module and then input a list of genes of the 19 interactors (ENSG00000108825 is not retrieved in the database) to obtain the data about the correlation of its expression.

Jvenn (http://bioinfo.genotoul.fr/jvenn) is an open-source component for web environments to process lists and produce Venn diagrams (Bardou et al., 2014). We pasted up our lists with one element per row to conduct an intersection analysis to compare the PPP1R14B-binding and interactive genes.

We used ShinyGO (http://bioinformatics.sdstate.edu/go/), a graphical gene set enrichment tool (Ge et al., 2020) for enrichment analysis by combining the PPP1R14B-correlated and interacted genes. Then we used the Gene Ontology chord plot tool in bioinformatics analyses (http://www.bioinformatics.com.cn/) to visualize the enrichment results.

### Statistical Analysis

Data about the association of PPP1R14B copy number alteration with its mRNA expression was obtained from the cBioPortal web (https://www.cbioportal.org/) and then analyzed using unpaired t-tests by GraphPad Prism (Version 8.0.1) for Windows. *P-*values less than 0.05 were considered statistically significant. The following annotations were used to show statistical significance: *p < 0.05, **p < 0.01, ***p < 0.001, and ****p < 0.0001.

## Results

### Expression of PPP1R14B in Human Pan-Cancer

**FIGURE 1:**
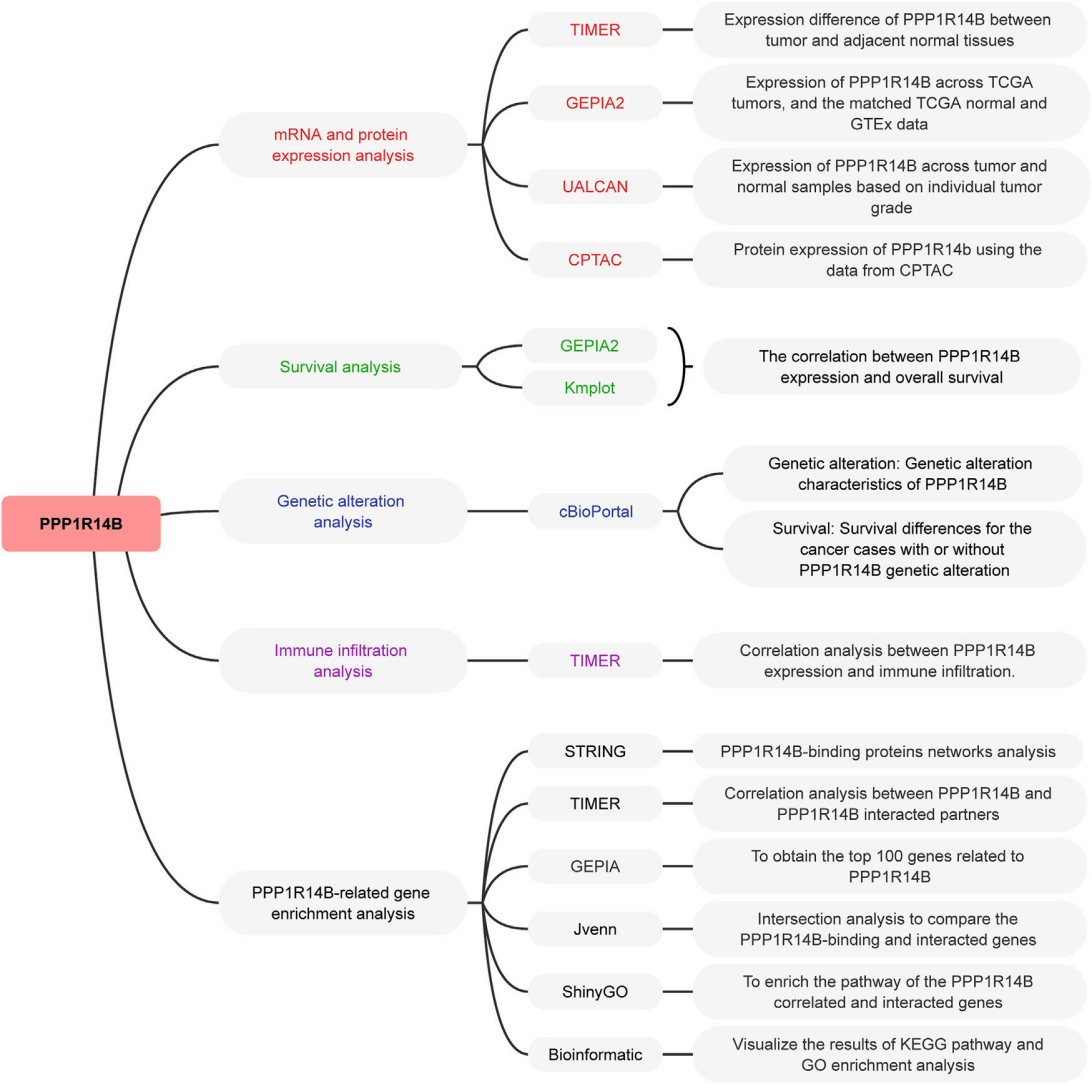
Setup of this study. The TCGA project and GEO databases were used to perform pan-cancer analysis of PPP1R14B.

We used the TCGA project and GEO databases to perform pan-cancer analysis of PPP1R14B, including expression differences, correlations between expression levels and survival, genetic alteration, immune infiltration, and relevant cellular pathways, to investigate the functions and potential mechanisms of PPP1R14B in the pathogenesis or clinical prognosis of different cancers. The setup of this study is shown in Figure 1.

**FIGURE 2:**
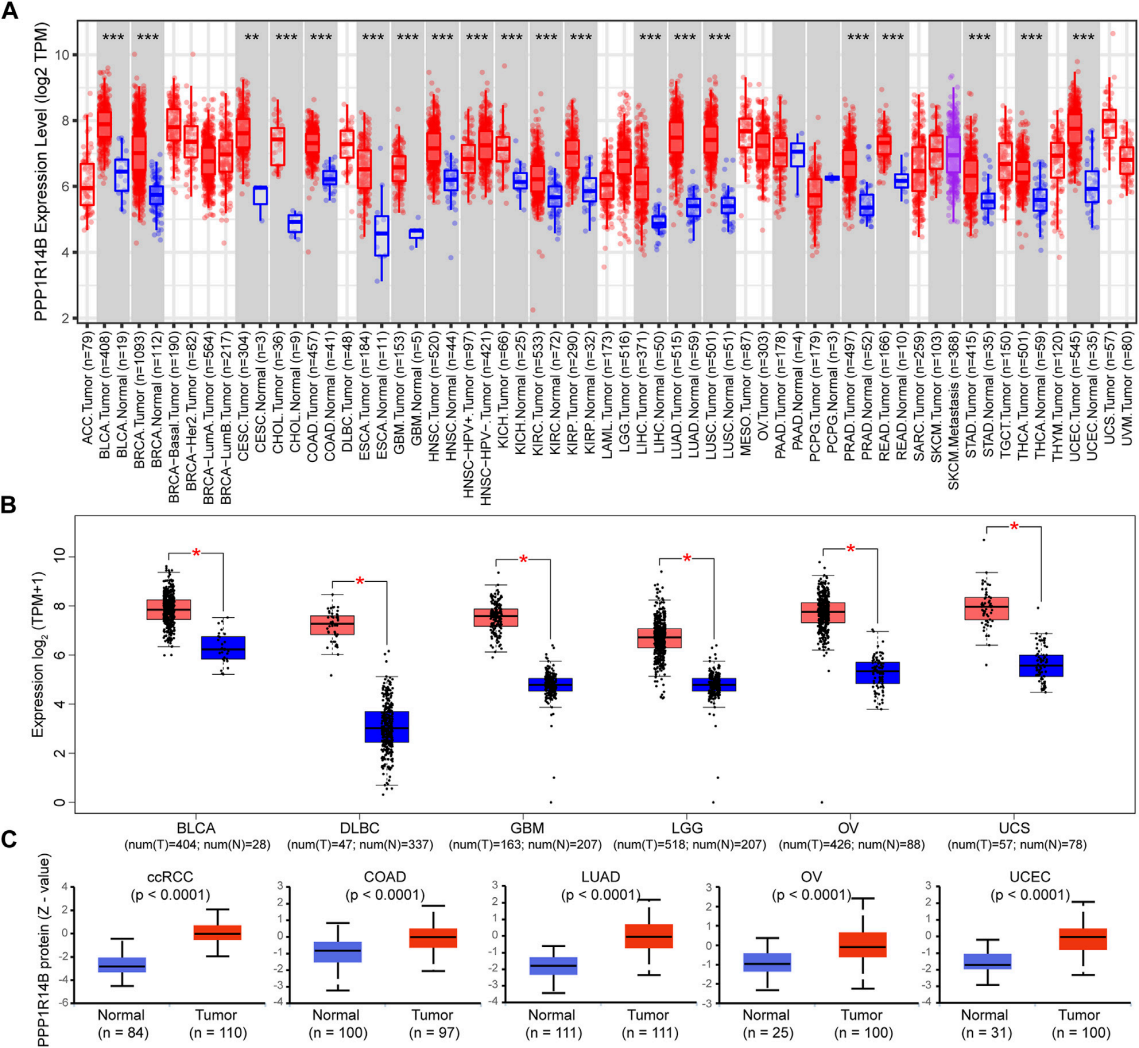
mRNA expression level of PPP1R14B in pan-cancer. **(A)** Human PPP1R14B expression levels in different cancer types from TCGA data in TIMER. **p < 0.01, ***p < 0.001 **(B)** For the type of BLCA, DLBC, GBM, LGG, OV, and UCS in the TCGA project, the normal tissues of the GTEx database were included as controls. The box plot data were supplied. **p < 0.01. **(C)** PPP1R14B proteomic expression profile in ccRCC, COAD, LUAD, OV, and UCEC from CPTAC samples. Z-values represent standard deviations from the median across samples for the given cancer type. N represents the number of samples.

To determine the differences of PPP1R14B expression between tumor and adjacent normal tissues, we used the TIMER2.0 database to analyze PPP1R14B mRNA expression levels across all TCGA tumors. The results showed that PPP1R14B was highly expressed in BLCA (bladder urothelial carcinoma), BRCA (breast invasive carcinoma), CESC (cervical squamous cell carcinoma and endo-cervical adenocarcinoma), CHOL (cholangio carcinoma), COAD (colon adenocarcinoma), ESCA (esophageal carcinoma), GBM (glioblastoma multiforme), HNSC (head and neck squamous cell carcinoma), KICH (kidney chromophobe), KIRC (kidney renal clear cell carcinoma), KIRP (kidney renal papillary cell carcinoma), LIHC (liver hepatocellular carcinoma), LUAD (lung adenocarcinoma), LUSC (lung squamous cell), PRAD (prostate adenocarcinoma), READ (rectum adenocarcinoma), STAD (stomach adenocarcinoma), THCA (thyroid carcinoma), and UCEC (uterine corpus endometrial carcinoma) compared with their adjacent normal tissues (Figure 2A).

In addition, considering that the TIEMR2.0 database lacks normal controls for some tumors, we chose the normal tissues of the GTEx dataset as controls to evaluate the expression difference of PPP1R14B between the normal and tumor tissues of BLCA (bladder urothelial carcinoma), DLBCL (lymphoid neoplasm diffuse large B-cell lymphoma), GBM (glioblastoma multiforme), LGG (brain lower grade glioma), OV (ovarian serous cystadenocarcinoma), and UCS (uterine carcinosarcoma). As shown in Figure 2B, PPP1R14B was highly expressed in the abovementioned tumor tissues compared with normal tissues.

Proteins are undoubtedly the main molecule related to disease, and the change in the protein expression level is directly related to disease, drug action, or toxin action. Therefore, we further analyzed the expression difference of the PPP1R14B protein between tumor and normal tissues using data from the Clinical Proteomic Tumor Analysis Consortium (CPTAC) (Ellis et al., 2013). The results showed that the PPP1R14B protein was highly expressed in ccRCC (Clear cell renal cell carcinoma), COAD, LUAD, OV, and UCEC (Uterine corpus endometrial carcinoma) compared with the normal tissues (Figure 2C).

### High Expression of PPP1R14B in Pan-Cancer on Different Stages

**FIGURE 3:**
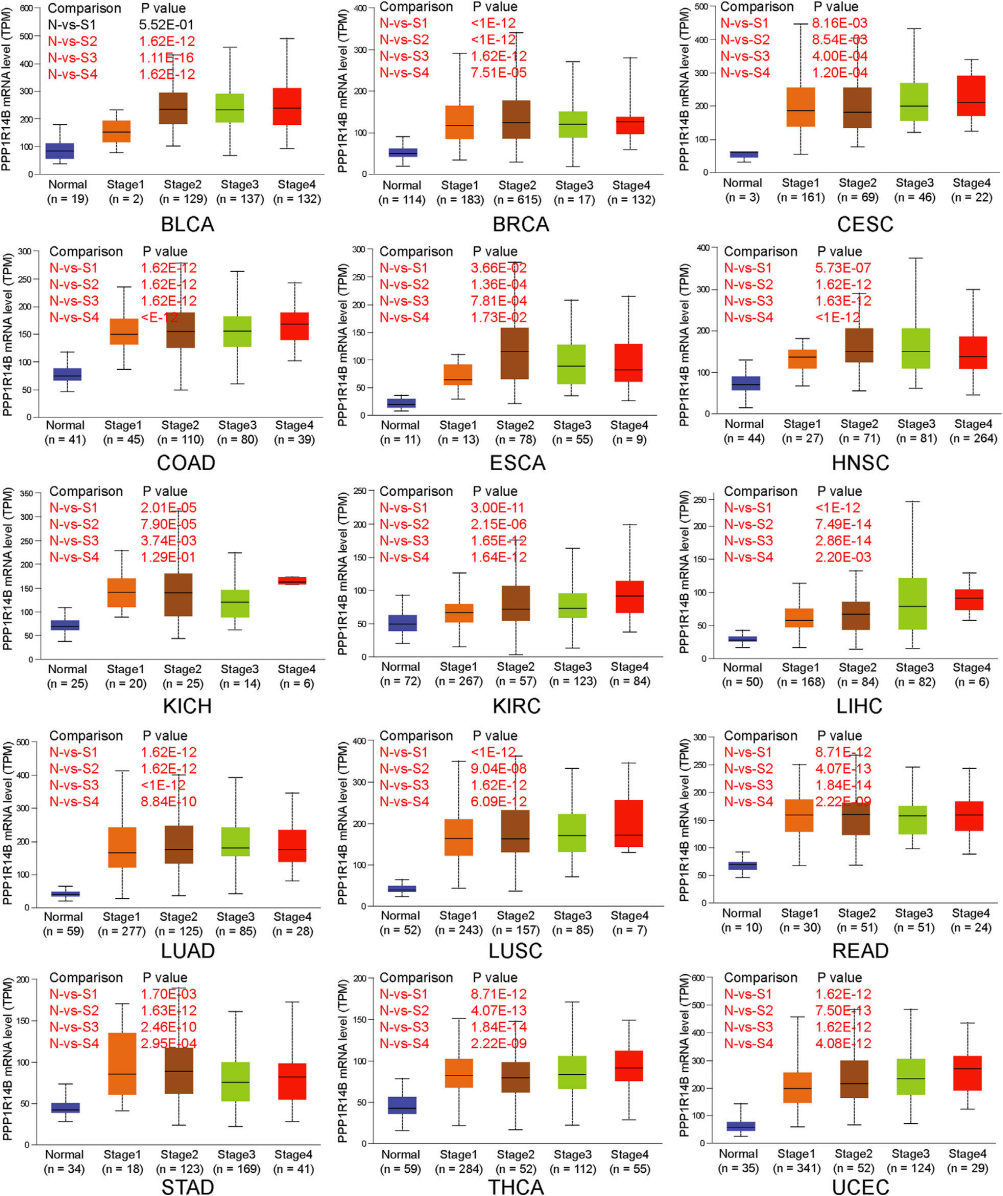
PPP1R14B overexpression in different pathologic stages. The expression of PPP1R14B according to the pathologic stage of the patient in the TCGA cancer type in the UALCAN database. X axis: pathologic cancer stages with the number of samples in each stage. Y axis: transcript per million. N, normal; S, stage. *p*-value marked red means the two groups are statistically significant.

Next, we investigated the expression of PPP1R14B according to the pathologic stage of the patients in the TCGA cancer type. We found that in BLCA, BRCA, CESC, COAD, ESCA, HNSC, KICH, KIRC, LIHC, LUAD, LUSC, READ, STAD, THCA, and UCEC, PPP1R14B expression levels were significantly higher in early stages. But there were no significant differences between the late stages and early stages. This indicates a possible involvement of PPP1R14B in the initiation but not the progression of cancer (Figure 3).

### High Levels of PPP1R14B Predict Poor Clinical Outcomes in Pan-Cancer

**FIGURE 4:**
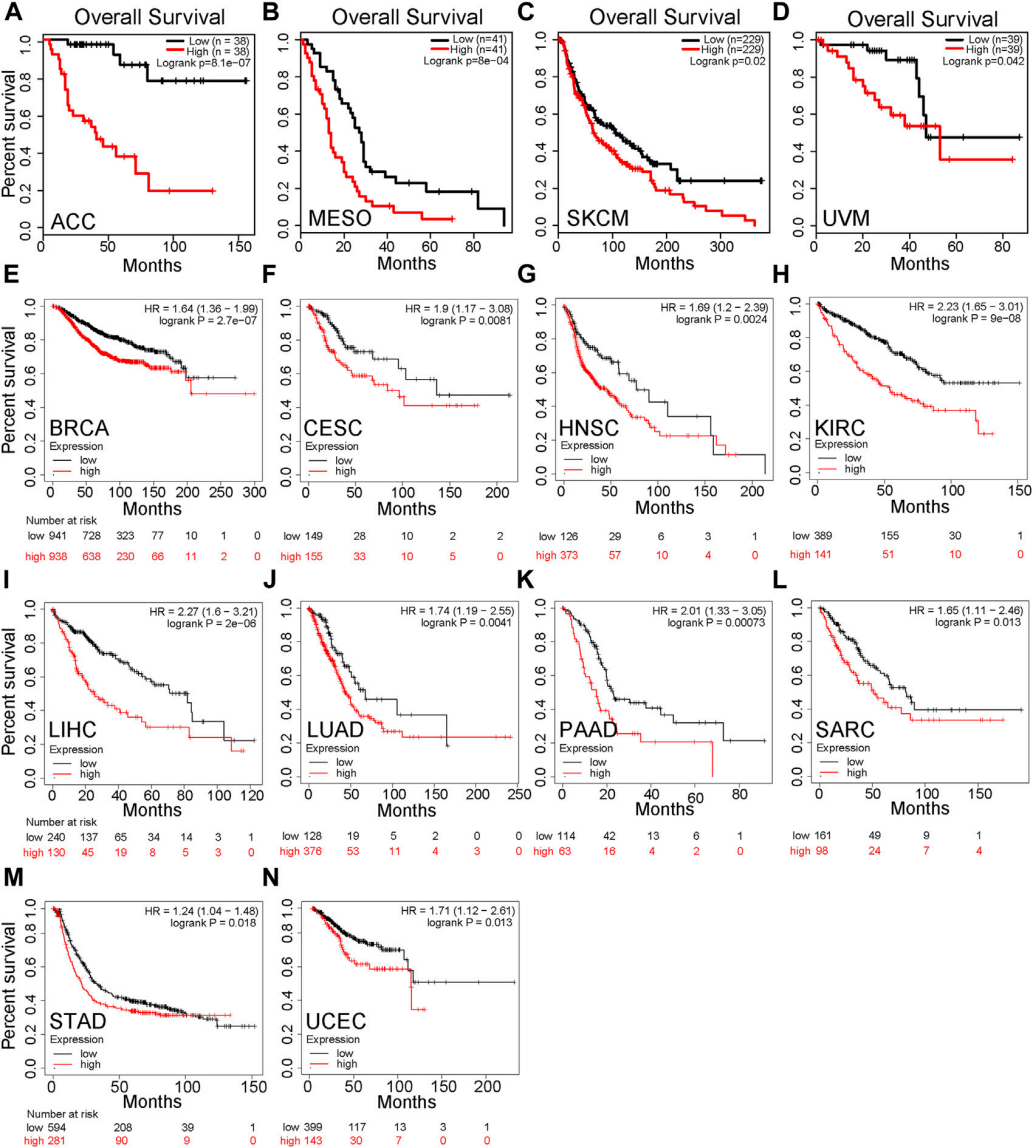
Correlation between PPP1R14B gene expression and survival prognosis of cancers in TCGA. **(A–D)** PPP1R14B expression and the prognosis of patients with different tumors in the TCGA and GEO datasets in GEPIA2. **(E–N)** Analysis of survival data using the Kaplan–Meier plotter tool.

We divided cancer cases into high-expression and low-expression groups according to the mRNA expression level of PPP1R14B and investigated the correlation between PPP1R14B expression and the prognosis of patients with different tumors in the TCGA and GEO datasets. These results showed that highly expressed PPP1R14B was linked to poor prognosis of overall survival (OS) for cancers of ACC (*p* = 8.1e-07), MESO (P = 8e-04), SKCM (*p* = 0.02), and UVM (*p* = 0.042) within the TCGA project in GEPIA2 (Figures 4A–D). In addition, the analysis of survival data using the Kaplan–Meier plotter tool showed a correlation between high expression of PPP1R14B and poor OS for BRCA (*p* = 2.7e-07), CESC (*p* = 0.0081), HNSC (*p* = 0.0024), KIRC (P = 9e-08), LIHC (2e-06), LUAD (*p* = 0.0041), PAAD (*p* = 0.00073), SARC (*p* = 0.013), STAD (*p* = 0.018), and UCEC (*p* = 0.013) within the TCGA project (Figures 4E–N). All of the abovementioned data showed that high expression of PPP1R14B was closely related to poor prognosis of most tumors, which may be a promising pan-cancer prognostic marker.

### Mutation Feature of PPP1R14B in Pan-Cancer

**FIGURE 5:**
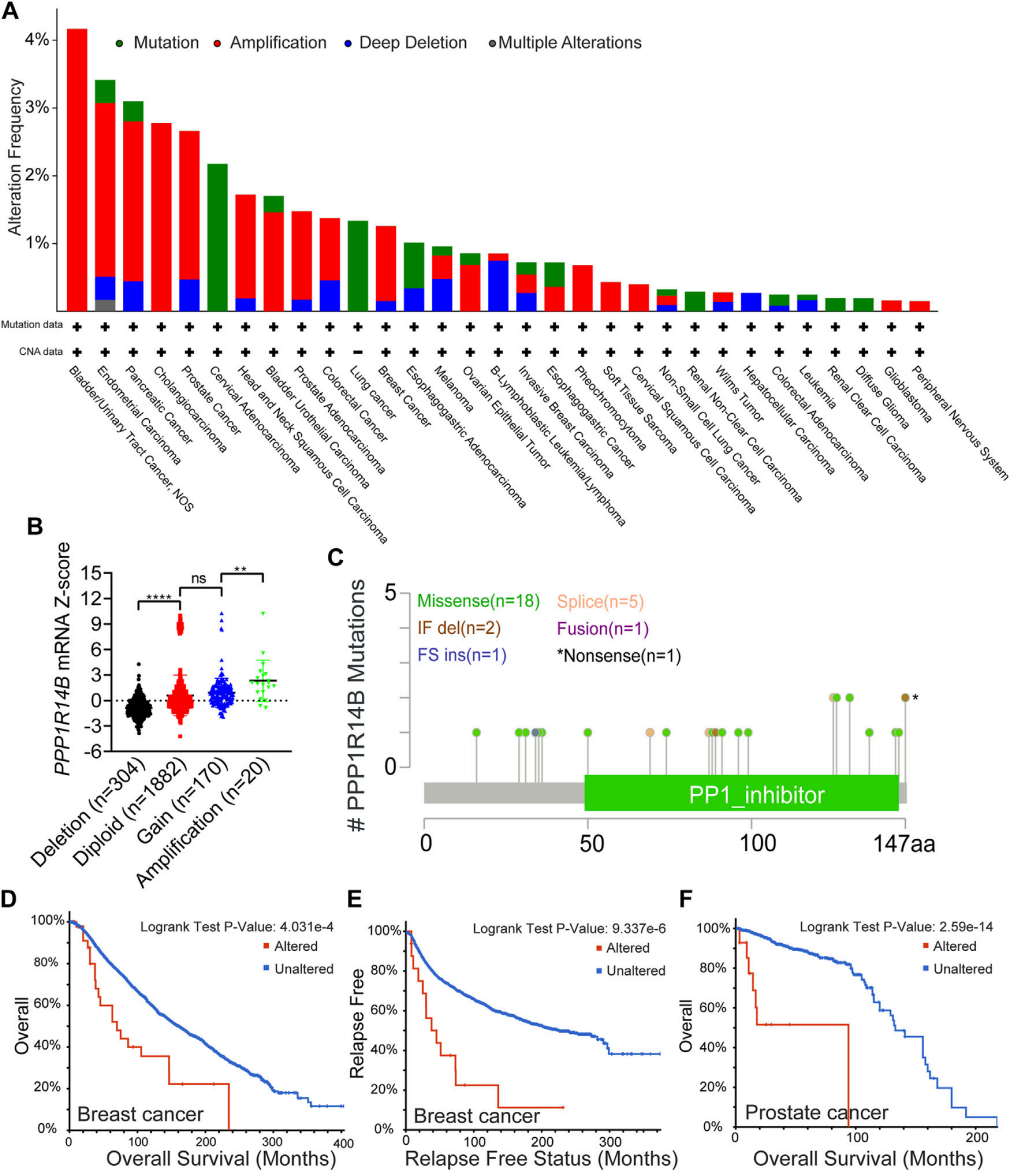
Mutation feature of PPP1R14B in pan-cancer. **(A)** Alteration frequency with the mutation type of PPP1R14B in human pan-cancer. **(B)** Association of PPP1R14B copy number alteration with its mRNA expression in the TCGA cancer cohort. **(C)** Sites and case numbers of the PPP1R14B genetic alteration were presented. **(D–F)** Association between genetic alteration of PPP1R14B and clinical survival.

In order to more systematically clarify the mutation feature and biological functions of PPP1R14B in tumor progression, we used the cBioPortal web to investigate the genetic alteration status of PPP1R14B in human pan-cancer. We observed that the highest alteration frequency of PPP1R14B (>4%) appears for patients with bladder/urinary tract cancer with “Amplification” as the primary type. In addition, the “amplification” type of CNA was also the primary type of genetic alteration in endometrial carcinoma, pancreatic cancer, cholangio carcinoma, and prostate cancer, of which the alteration frequency exceeded 2%. We also observed that the “mutation” type of CNA was the primary alteration type in cervical adenocarcinoma and lung cancer with an alteration frequency of 1–2% (Figure 5A). Then, we retrieved the PPP1R14B expression data from TCGA pan-cancer dataset and found that PPP1R14B was amplified along with the significantly high mRNA expression (Figure 5B). The types, sites, and case numbers of the PPP1R14B genetic alteration are further presented in Figure 5C. We found that the missense mutation of PPP1R14B was the main type of genetic alteration, which was detected in 18 cases. In addition, five cases contained the splice mutation and two cases carried “In_Frame_Del” (IF del) mutation. The fusion, FS ins, and nonsense mutations were detected in one case alone. Additionally, we explored the association between genetic alteration of PPP1R14B and clinical survival in different cancer. As shown in Figures 5D,E, the BRCA cases with altered PPP1R14B showed poor OS (*p* = 4.031e-04) and relapse-free survival (*p* = 9.337e-06). The prostate cancer cases with altered PPP1R14B also showed poor OS (*p* = 2.59e-14) (Figure 5F). These results suggested that genetic alteration of PPP1R14B in tumors is involved in PPP1R14B mRNA expression alteration and poor clinical survival, which deserved more in-depth research.

### Correlation Analysis Between PPP1R14B Expression and Immune Infiltration of MDSCs

**FIGURE 6:**
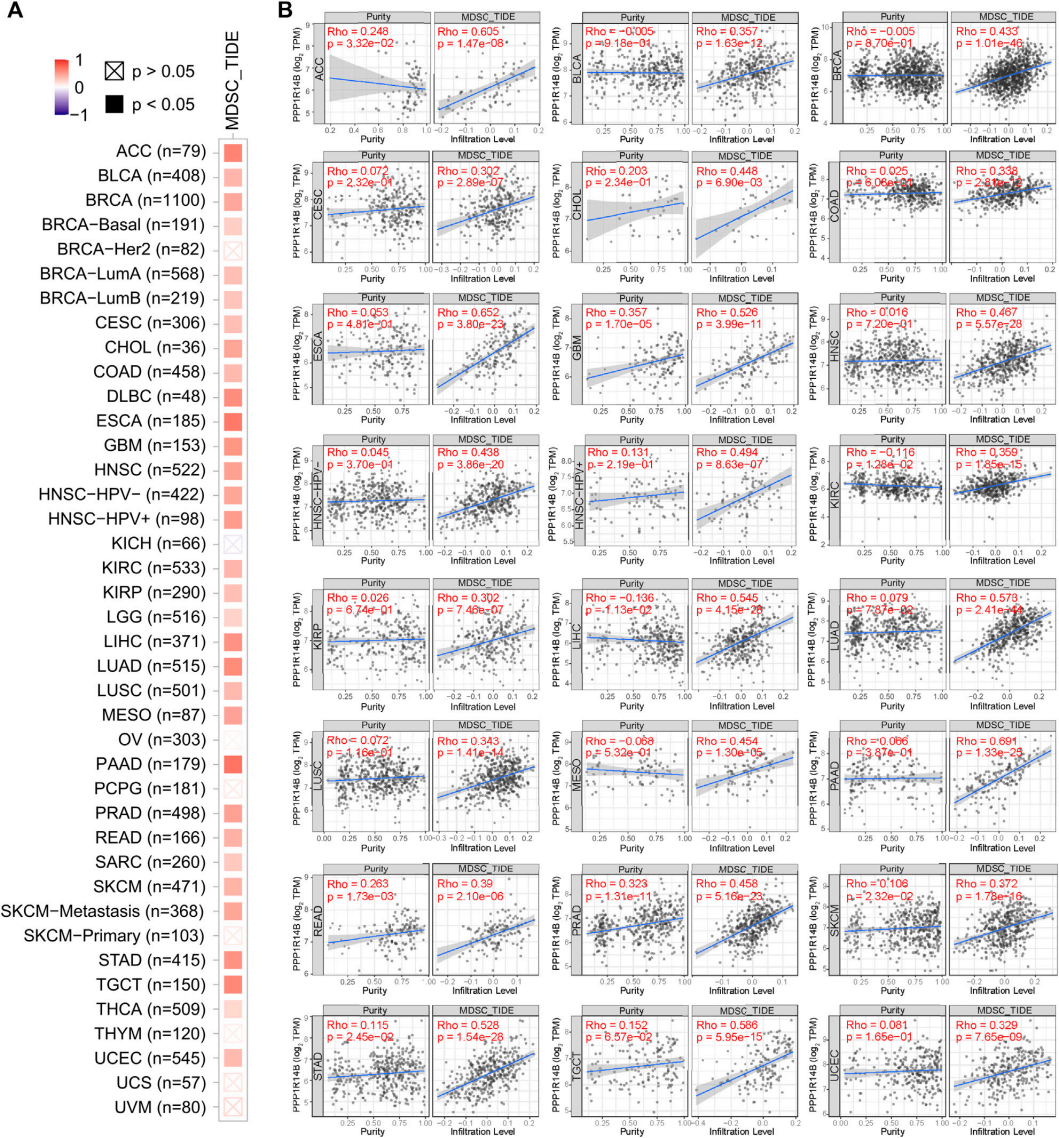
Correlation analysis between PPP1R14B expression and MDSC infiltration. Heatmap **(A)** and scatter plot **(B)** represented the relationship between MDSC infiltration and PPP1R14B gene expression across all types of cancer in TCGA.

The level of immune infiltration in the TME is closely correlated with the initiation, progression, or metastasis of cancer. We investigated the various immune cell infiltration levels in diverse cancer types in the TIMER database. As shown in Supplementary Table S1, the PPP1R14B expression level was significantly correlated with MDSC infiltration in BLCline 277-280A, BRCA, CESC, LIHC, LUAD, PAAD, PRAD, STAD, and THCA. However, there was no universally significant correlation between PPP1R14B expression and the other subgroup immune cell infiltrations, which included B cells, CD4^+^ T cells, CD8^+^ T cells, Tregs, Tfh cells, γδ T cells, monocytes, macrophages, neutrophils, and DC. Therefore, we focused on the MDSC in the TME. As shown in Figure 6A, the heatmap and scatter plot presented the relationship between the infiltrate estimation value and PPP1R14B gene expression. The results showed that in most cancer types, the expression of PPP1R14B is positively correlated with the MDSC infiltration level (Figure 6B). Furthermore, we investigated the correlation of the MDSC infiltration level and the prognosis of patients with different tumors in TCGA datasets in TIMER2.0. These results suggested that high levels of MDSC infiltration predict poor clinical outcomes in pan-cancer (Supplementary Figure S1). Our studies suggested that PPP1R14B is involved in the tumor immunology process.

### PPP1R14B-Related Gene Enrichment Analysis

**FIGURE 7:**
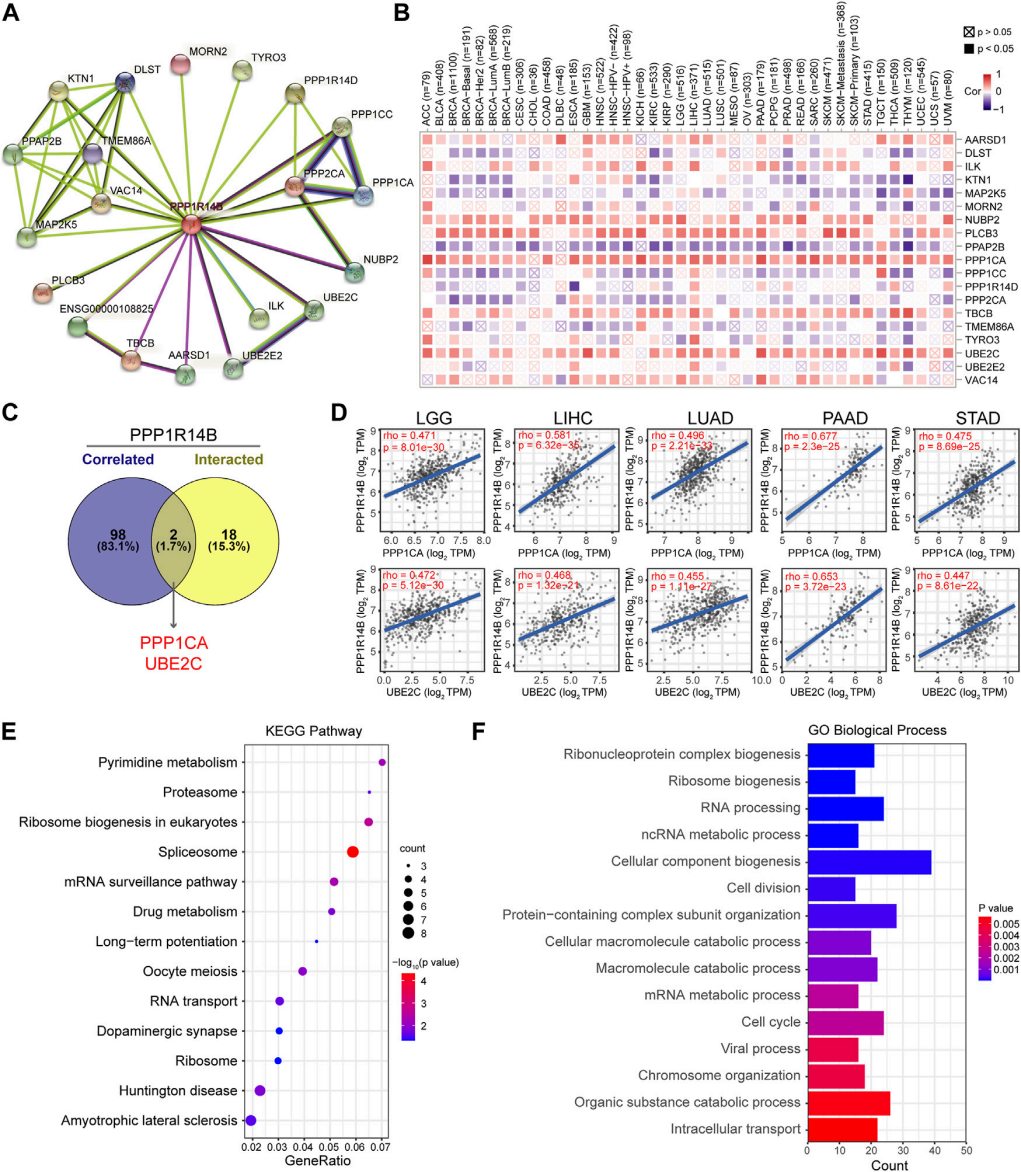
PPP1R14B-related gene enrichment analysis. **(A)** PPP1R14B interaction network analysis. **(B)** Correlation of PPP1R14B with the 20 interactors of PPP1R14B-binding. **(C)** Intersection analysis of PPP1R14B-correlated genes and PPP1R14B-interacted partners. **(D)** Correlations of PPP1R14B with PPP1CA and UBE2C. **(E)** KEGG enrichment analysis based on the PPP1R14B-correlated and interacted genes. **(F)** GO analysis based on the PPP1R14B-binding and interacting genes.

To further study the molecular mechanism of PPP1R14B in tumorigenesis, we tried to screen out PPP1R14B-binding proteins for protein–protein interaction network analysis. Using the STRING online tool, we obtained a total of 20 PPP1R14B binding proteins, including members which were supported by experimental evidence or were predicted (Figure 7A). Then, we analyzed the correlation of PPP1R14B with the 20 interactors of PPP1R14B-binding in various cancer types. As shown in Figure 7B, the expression of NUBP2, PLCB3, PPP1CA, TBCB, and UBE2C were positively correlated with PPP1R14B in the majority of cancer types. Then, we used the GEPIA2 tool to combine all tumor expression data of TCGA and obtained the top 100 genes related to the expression of PPP1R14B. Through intersection analysis of the abovementioned two groups, we obtained two common members, namely, PPP1CA and UBE2C (Figure 7C). PPP1CA and UBE2C have strong positive correlations with PPP1R14B expression (Figure 7D).

In addition, we used the ShinyGO tool for enrichment analysis based on the two sets of data. Here, we performed Kyoto Encyclopedia of Genes and Genomes (KEGG) pathway analysis and Gene Ontology (GO) enrichment analysis. Then, the Gene Ontology chord plot tool in was used in bioinformatics to visualize the enrichment results. The KEGG pathway analysis data further indicated that PPP1R14B may be involved in oncogenesis through the “pyrimidine metabolism”, “ribosome biogenesis in eukaryotes”, “spliceosome” and “mRNA surveillance pathway” (Figure 7E). The GO enrichment analysis suggested that these genes were mainly related to “cellular component biogenesis”, “RNA processing”, “cell cycle”, “cell division”, and “ribonucleoprotein complex biogenesis” biological processes (Figure 7F).

## Discussion

PPP1R14B encodes a putative inhibitor of protein phosphatase 1 (PP1), a pleiotropic enzyme which plays a multifaceted role in cellular growth, cell cycle regulation, and apoptosis (Figueiredo et al., 2014). The recent studies showed that SCGB2A1, KLF4, and PPP1R14B can differentiate a group of circa 5% of cases with less survival in chronic lymphocytic leukemia (CLL) patients, which might be useful for further research about disease prognostication and drug response in CLL (Orgueira et al., 2019). PPP1R14B was significantly overexpressed in OCCC and associated endometriosis (Worley et al., 2015) and overexpressed in plasma messenger RNAs in PCa (prostate cancer) patients (Wang et al., 2020), but the role of PPP1R14B in cancer was still unknown.

In our study, we found that PPP1R14B was highly expressed in most types of tumors, and there were significant differences in early stages, which indicate a possible involvement of PPP1R14B in the initiation of cancer. In addition, patients with a high level of PPP1R14B predicted poor clinical outcomes in pan-cancer.

Mutation feature analysis of PPP1R14B in pan-cancer suggested that the highest alteration frequency of PPP1R14B (>4%) appears for patients with bladder/urinary tract cancer with “Amplification” as the primary type, and the genetic alteration of PPP1R14B in tumors changed its mRNA expression. Prostate cancer and breast cancer cases with altered PPP1R14B showed poorer clinical survival compared with the unaltered PPP1R14B group.

The infiltration of immune cells is a hallmark of most forms of malignancy. The level of immune infiltration in the TME is closely correlated with the initiation, progression, or metastasis of cancer. The TCGA database helps to find associations between immune infiltration, gene expression, mutations, and survival features in the TCGA cohorts (El-Arabey et al., 2020). The TCGA database can be used for the characterization of the prevalence and variability of Tumor Inflammation Signature (TIS) and understanding the immune status of tumors, to improved indication selection for testing immunotherapy agents. Identified key changes in immune cells in the TME help improve the development of treatments (Danaher et al., 2018). Here, we found that the PPP1R14B expression level was positively correlated with MDSC infiltration in many cancer types. The accumulation of the relatively immature and pathologically activated MDSC with potent immunosuppressive activity is common in tumors. The MDSC promotes tumor progression by promoting tumor cell survival, angiogenesis, invasion, and metastasis (Condamine et al., 2015b) and plays a major role in the negative regulation of the immune response in cancer (Condamine et al., 2015a). MDSCs not only inhibited a variety of immune cells (including T cells and NK cells) but also are involved in tumor progression by inducing epithelial–mesenchymal transition (EMT), tumor angiogenesis, tumor cell invasion, formation of pre-metastatic niches, and cancer stem cells (CSCs) (Condamine et al., 2015b). MDSC infiltration levels are closely associated with clinical outcomes and therapeutic effects (Zhang et al., 2016). MDSCs promoting tumor progression have been demonstrated in several animal models (Talmadge and Gabrilovich, 2013). MDSCs are attracted to tumor sites in response to various different cytokines. Two major subsets of MDSCs have been identified so far: monocytic (M-MDSC) and polymorphonuclear (PMN-MDSC). The M-MDSC is attracted to tumor sites by CCL2, CCL5, and CSF1 and PMN-MDSC was attracted by CXCL1, CXCL5, CXCL6, CXCL8, and CXCL12 (Kumar et al., 2016). A variety of growth factors secreted by many tumor cells, such as CSF1, CSF2, and VEGF, are also involved in regulating the fate of the MDSC in the TME (Gabrilovich et al., 2012). However, how the elevated expression of PPP1R14B in tumor tissues induces an increase of the MDSC infiltration level should be further investigated. The recruitment of chemokines, the clonal expansion of MDSCs in a complex microenvironment, and proliferation and apoptosis will affect the number and functions of MDSCs in the TME. The regulation of PPP1R14B on the TME is a complex problem, and information from our existing database will be insufficient to clarify the mechanism. Single-cell RNA-seq (scRNA-seq) has been widely used to dissect the composition of the TME in various cancer indications (Giladi and Amit, 2018; Hornburg et al., 2021). With tumor profiling data from single-cell technologies being increasingly available, there will be more precise understanding of the characterization of tumor-infiltrating immune cells and potential molecular mechanisms that shape the tumor immune phenotypes.

Furthermore, in order to reveal the mechanism of PPP1R14B in tumor progression, our study identified co-expression genes associated with the PPP1R14B protein network. These results showed that PPP1CA and UBE2C had strong positive correlations with PPP1R14B expression in most cancers. PPP1CA is reported to be one of three catalytic subunits of protein phosphatase 1 (PP1) and has been shown to be closely related to the development of malignant tumors (Chen et al., 2018). The USP11/PPP1CA complex promoted CRC progression by activating the ERK/MAPK signaling pathway (Sun et al., 2019). UBE2C, the ubiquitin-conjugating enzyme, is overexpressed in all 27 cancers. Patients with higher UBE2C levels showed a shorter overall survival (OS) time and worse disease-free survival prognosis (DFS) (Dastsooz et al., 2019). Our data highlighted that the PPP1R14B network was one of the main protein networks related to cancer, and further study of its function in tumors may provide clues for new cancer treatment strategies.

However, even though we integrated information across multiple databases about the role of PPP1R14B in pan-cancer, this study still had limitations. Because the microarray and sequencing data about PPP1R14B were collected by analyzing tumor tissue information, the immune cell marker analysis could have introduced systematic bias. Also there is no information about posttranslational modification of PPP1R14B, which may influence its function. In addition, this study only conducted a bioinformatics analysis concerning the role of PPP1R14B in pan-cancer across different databases, and *in vivo* and *in vitro* experiment verification and further mechanism research is also needed. Cancer evolution includes the complex interaction of heredity, cell state, epigenetic, spatial, and microenvironmental factors. With the rapid development of sequencing and omics technology, multi-omics, single-cell multi-omics, and spatial transcriptomics will add wings to the investigation of the complex environment of tumors. Integrated characterization and analysis using transcriptomic, proteomic, and metabolomic molecular profiles of tumor patients could identify the key pathways and metabolites, which is more accurate than a single transcriptomic analysis (Su et al., 2020). Integrating multiple layers of information from single-cell multi-omics is essential for a comprehensive understanding of the mechanism of cancer evolution (Nam et al., 2021). However, single-cell sequence experiments need to prepare the tissue into a single-cell suspension, which loses the original location information of the cells in the tissue, and also breaks the communication network between cells. Therefore, it is difficult to obtain the cell composition and structure of different regions in the tissue, information about gene expression status, and gene differential expression between different functional regions (Stahl et al., 2016). Spatial transcriptomic analysis can fully reveal the complexity of the TME and explore tumor occurrence and development from a multidimensional perspective of time and space (Berglund et al., 2018). With the rapid development of single-cell omics technology, there are more and more single-cell omics data. At the same time, the birth of new algorithms and models has also improved the accuracy of the integration and analysis of single-cell omics datasets, such as single-cell Graph Convolutional Network (scGCN) (Song et al., 2021), deconvoluting spatial transcriptomics data through Graph-Based Convolutional Networks (DSTG) (Song and Su, 2021) and single-cell latent variable model (scLM) (Song et al., 2020). Multi-omics, as well as the advanced technology such as single-cell multi-omics and spatial transcriptomics have great potential in investigating underlying roles of PPP1R14B in the TME and multidimensional space-time perspective to explore the role of PPP1R14B in the occurrence and development of tumors.

In summary, PPP1R14B can affect the prognosis of pan-cancer and is closely related to immune infiltration. Increased PPP1R14B expression correlates with poor prognosis and increased immune infiltration levels in the MDSC. PPP1R14B can be used as a prognostic biomarker for pan-cancer. These findings may provide an antitumor strategy targeting PPP1R14B, including manipulation of tumor cell growth or the TME, especially MDSC infiltration.

## Data Availability

The datasets presented in this study can be found in online repositories. The names of the repository/repositories and accession number(s) can be found in the article/Supplementary Material.

## Glossary

TCGA: The Cancer Genome Atlas
CPTAC: Clinical Proteomic Tumor Analysis Consortium
KEGG: Kyoto Encyclopedia of Genes and Genomes
GO: Gene Ontology
BLCA: Bladder urothelial carcinoma
BRCA: Breast invasive carcinoma
PCa: Prostate cancer
CESC: Cervical squamous cell carcinoma and endo-cervical adenocarcinoma
CHOL: Cholangio carcinoma
COAD: Colon adenocarcinoma
ESCA: Esophageal carcinoma
GBM: Glioblastoma multiforme
HNSC: Head and neck squamous cell carcinoma
KICH: Kidney chromophobe
KIRC: Kidney renal clear cell carcinoma
KIRP: Kidney renal papillary cell carcinoma
LIHC: Liver hepatocellular carcinoma
LUAD: Lung adenocarcinoma
LUSC: Lung squamous cell
PRAD: Prostate adenocarcinoma
READ: Rectum adenocarcinoma
STAD: Stomach adenocarcinoma
THCA: Thyroid carcinoma
UCEC: Uterine corpus endometrial carcinoma
DLBC: Lymphoid neoplasm diffuse large B-cell lymphoma
GBM: Glioblastoma multiforme
LGG: Brain lower grade glioma
OV: Ovarian serous cystadenocarcinoma
UCS: Uterine carcinosarcoma
OCCC: Ovarian clear cell carcinoma
OS: Overall survival
DFS: Disease-free survival prognosis
ccRCC: Clear cell renal cell carcinoma
