# Supplementary material for: PPP1R14B Is a Prognostic and Immunological Biomarker in Pan-Cancer
Source: Front Genet. 2021 Nov 11;12:763561. doi: 10.3389/fgene.2021.763561 (PMC8631915; doi:10.3389/fgene.2021.763561)
Supplement: Supplementary file 1 [file DataSheet1.doc]

**PPP1R14B Is a Prognostic and Immunological**

**Biomarker in Pan-Cancer**

# Supplementary data

# Correlation analysis between PPP1R14B expression and immune infiltration.

# We investigated the various immune cells infiltration level in diverse cancer types in the TIMER database. As shown in Table S1, the results revealed the PPP1R14B expression level was significantly correlated with MDSC infiltration in BLCA, BRCA, CESC, LIHC, LUAD, PAAD, PRAD, STAD and THCA. However, there was no universally significant correlation between PPP1R14B expression and the other subgroup immune cells infiltration, which included B cells, CD4+ T cells, CD8+ T cells, Treg, Tfh, γδ T cells, monocytes, macrophage, neutrophil, and DC.

**Table S1 | Correlation analysis between PPP1R14B expression and immune infiltration.**

| Cell Type | Description | BLCA (n=408) | BRCA (n=1100) | CESC (n=306) | ESCA (n=185) | LIHC (n=371) | LUAD (n=515) | PAAD (n=179) | PRAD (n=498) | STAD (n=415) | THCA (n=509) |
| --- | --- | --- | --- | --- | --- | --- | --- | --- | --- | --- | --- |
| B cell | Cox | -0.19 | -0.03 | -0.25 | -0.46 | 0.05 | -0.16 | -0.32 | -0.30 | -0.19 | -0.24 |
|  | *p* | *** | ns | *** | *** | ns | *** | *** | *** | *** | *** |
| CD4 | Cox | -0.11 | -0.40 | -0.04 | 0.27 | -0.18 | -0.38 | -0.32 | -0.61 | -0.44 | -0.29 |
|  | *p* | * | *** | ns | *** | *** | *** | *** | *** | *** | *** |
| CD8 | Cox | -0.16 | -0.21 | -0.33 | -0.22 | 0.06 | -0.22 | 0.00 | -0.49 | -0.14 | -0.14 |
|  | *p* | ** | *** | *** | ** | ns | *** | ns | *** | ** | ** |
| Treg | Cox | -0.04 | 0.27 | 0.04 | -0.57 | 0.26 | 0.11 | 0.22 | 0.32 | 0.23 | 0.21 |
|  | *p* | ns | *** | ns | *** | *** | * | ** | *** | *** | *** |
| Tfh | Cox | 0.03 | 0.28 | -0.02 | 0.02 | 0.14 | 0.15 | 0.16 | 0.07 | 0.27 | -0.01 |
|  | *p* | ns | *** | ns | ns | * | ** | * | ns | *** | ns |
| γδ T | Cox | -0.08 | -0.07 | 0.02 | 0.00 | -0.06 | -0.01 | -0.11 | 0.11 | -0.12 | -0.04 |
|  | *p* | ns | * | ns | ns | ns | ns | ns | * | * | ns |
| Monocyte | Cox | -0.09 | 0.07 | 0.06 | 0.08 | 0.28 | -0.07 | -0.22 | -0.03 | -0.21 | -0.10 |
|  | *p* | * | * | ns | ns | *** | ns | ** | ns | *** | * |
| Macrophage | Cox | -0.21 | -0.25 | -0.17 | -0.23 | 0.18 | -0.14 | -0.29 | -0.24 | -0.29 | -0.43 |
|  | *p* | *** | *** | ** | ** | *** | ** | *** | *** | *** | *** |
| Neutrophil | Cox | -0.20 | -0.47 | -0.11 | 0.11 | -0.33 | -0.19 | -0.02 | -0.17 | -0.16 | -0.17 |
|  | *p* | *** | *** | ns | ns | *** | *** | ns | *** | ** | *** |
| DC | Cox | -0.17 | -0.10 | -0.26 | -0.11 | 0.11 | -0.33 | -0.39 | -0.23 | -0.36 | -0.13 |
|  | *p* | *** | *** | *** | ns | * | *** | *** | *** | *** | ** |
| MDSC | Cox | 0.36 | 0.43 | 0.30 | 0.65 | 0.55 | 0.57 | 0.69 | 0.46 | 0.53 | 0.20 |
|  | *p* | *** | *** | *** | *** | *** | *** | *** | *** | *** | *** |

Treg, regulation T cell; Tfh, follicular helper T cell; DC, dendritic cell; MDSC, Myeloid derived suppressor cell; Cox, R value of Spearman’s correlation. * p < 0.05, ** p < 0.01, *** p < 0.001, ns, no significant differences.

# High levels of MDSC infiltration predicts poor clinical outcomes in pan-cancer

Our results show that the expression of PPP1R14B are positively correlated with the level of MDSC infiltration in most cancer types. So we further investigate the influence of MDSC infiltration on clinical outcomes of cancer. The results suggest that in most types of tumors, patients with high levels of MDSC infiltration have poor survival (Figure S1).

**
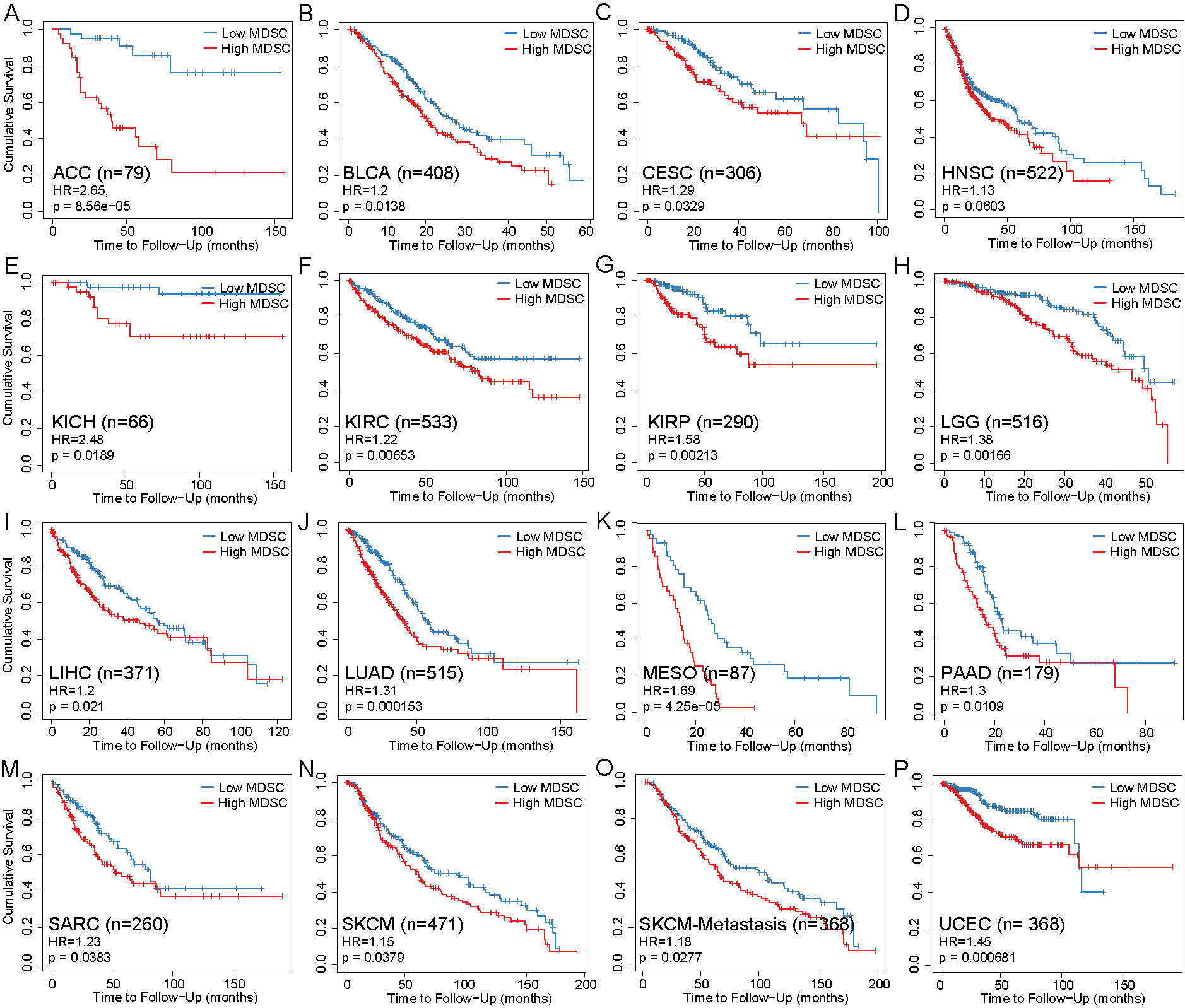
**

**FIGURE S1 | High levels of MDSC infiltration predicts poor clinical outcomes in pan-cancer.**

**(A-P)** Correlation of MDSC infiltration level and the prognosis of patients with different tumors in TCGA data sets in TIMER2.0.
